# BLyS/APRIL dual inhibition for IgG4-RD: a prospective single-arm clinical trial of telitacicept

**DOI:** 10.1136/ard-2022-223529

**Published:** 2023-01-19

**Authors:** Shaozhe Cai, Ziwei Hu, Yu Chen, Yuxue Chen, Bingxia Ming, Rongfen Gao, Zhen Li, Cong Ye, Jixin Zhong, Lingli Dong

**Affiliations:** 1 Department of Rheumatology and Immunology, Tongji Hospital, Tongji Medical College, Huazhong University of Science and Technology, Wuhan, Hubei, China; 2 Department of Radiology, Tongji Hospital, Tongji Medical College, Huazhong University of Science and Technology, Wuhan, Hubei, China

**Keywords:** Glucocorticoids, B-Lymphocytes, Autoimmune Diseases

IgG4-related disease (IgG4-RD) is a newly recognised fibro-inflammatory clinical entity with multiorgan involvement. Although most patients with IgG4-RD respond well to glucocorticoids, they have limited treatment options for disease control and high relapse rates were reported during long-term treatment.^1^ We report here a series of IgG4-RD cases treated with telitacicept, a novel bioagent (TACI-immunoglobin fusion protein) simultaneously targeting B cell maturation signals BLyS (BAFF) and APRIL,^2^ in the absence of long-term glucocorticoids.

**Figure 1:**
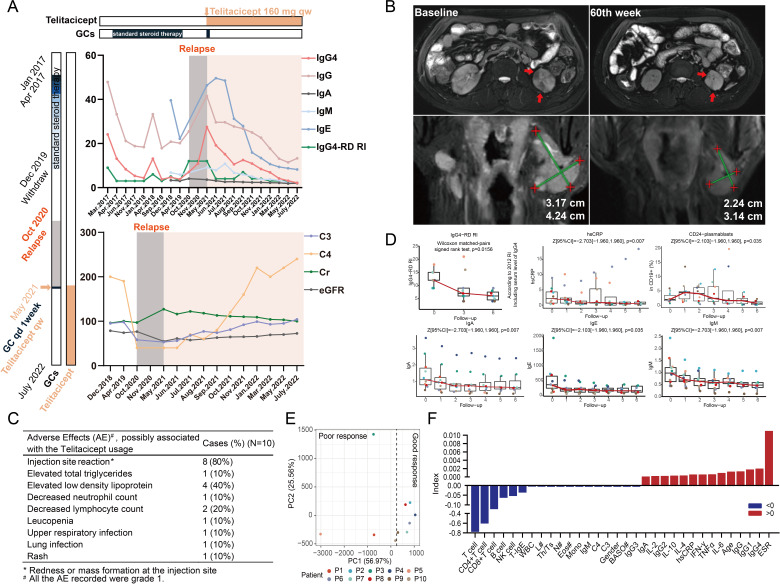
Clinical and laboratory parameters in IgG4-related disease (IgG4-RD) treated with telitacicept. (A) Left part shows previous IgG4-RD treatment strategy. The patient was diagnosed with IgG4-RD in Tongji Hospital in April 2017. At this point, the patient was treated with oral prednisone (30 mg orally) for 2 months. Prednisone dosage decreased regularly by 5 mg every 2 or 3 months to 5 mg in April 2018, which was maintained for 20 months until December 2019. And then withdrawal of prednisone sustained for 17 months until May 2021. (A) Right part shows serum levels of IgG (g/L), IgM (*10 g/L), IgE (*0.1 IU/L), IgA (g/L), IgG1-4 (g/L), C3 (g/L), C4 (g/L), Cr (µmol/L), eGFR (ml/min/1.73m^2^) and IgG4-RD RI before and after treatment with telitacicept. Pink areas indicate the period of telitacicept treatment. Bar plot on the left and upper area shows the timeline of treatment from the first onset of IgG4-RD. (B) The maximum lesion area of left submandibular gland and lymph node in coronal plane, and the largest maximum lesion area of kidneys (red arrows) in transverse plane before and after 60 weeks of telitacicept treatment. (C) The safety data recorded during the telitacicept treatment. (D) Mann-Kendall trend analyses of the IgG4-RD RI, CD24-plasmablasts, IgE and IgM during the follow-up. (E) Principal component analysis of the baseline immune laboratory data of the enrolled cases. (F) Analysis of the coefficients of x-variables of the PC1’s linear combination with the following baseline indices: gender, age, WBC, N#, L#, Mono#, Eos#, Baso#, ESR, hsCRP, IgG, IgA, IgM, C3, C4, IgG1, IgG2, IgG3, IgG4, IgE, IL-2, IL-4, IL-6, IL-10, TNF-α, IFN-γ, T cell, CD8+T cell, CD4+T cell, NK cell, B cell, Th/Ts. Data were analysed and plotted with Prism software (V.8.0.2) or R software (V.4.1.1). Baso#, absolute basophil count; Eos#, absolute eosinophil count; ESR, erythrocyte sedimentation rate; hsCRP, high-sensitive C-reactive protein; L#, absolute lymphocyte count; N#, absolute neutrophil count; Mono#, absolute monocyte count; RI, Responder index Index; WBC, white blood cell.

A 54-year-old man, who was diagnosed with IgG4-RD in 2017, presented in our department with re-enlarged bilateral submandibular glands, parotid glands and 12 lymph nodes after withdrawal of glucocorticoids (with a new onset of renal involvement). His 2019 American College of Rheumatology/European League Against Rheumatism classification criteria (ACR/EULAR)^3^ classification criteria score was 41 and IgG4-RD Responder Index (RI)^4^ was 12 at admission. After the discontinuation of glucocorticoids within 1 week, we initiated the treatment of telitacicept (160 mg every week hypodermic injection) for 60 weeks due to his unwillingness to use long-term systemic glucocorticoid therapy. His symptoms were gradually relieved after the initiation of treatments. IgG4-RD RI decreased from 12 to 1 at week 60 (figure 1A). The levels of IgG4, IgE, IgG and IgM decreased and serum complement C3, C4, creatinine and eGFR returned to normal during the treatment of telitacicept (figure 1A). MRI detections showed a gradual and persistent reduction in the sizes of the involved salivary glands (returned to normal size at week 60) and the renal cortex lesions (almost diminished) during the 60 weeks’ treatment with telitacicept (figure 1B and online supplemental figure S1).

10.1136/ard-2022-223529.supp1 Supplementary data

Besides the aforementioned case, based on the criteria listed in online supplemental table S1, we recruited additional nine IgG4-RD cases (eight belonged to the ‘Mikulicz and Systemic’ group and one belonged to the ‘Head and Neck-Limited’ group,^5^ online supplemental table S2) and treated with the same therapeutic strategy to examine the remission rate after telitacicept treatment. No severe adverse event was observed, however, injection site reactions (redness or mass formation), which were mild and controllable, were observed in 80% of the patients (figure 1C). Similar high ratio of injection site reactions of telitacicept administration could also be observed in clinical trials for other diseases.^6^ Trend analyses showed significant decreases in IgG4-RD RI, serum IgM, IgE and CD19^+^CD24^-^CD38^hi^ plasmablast levels during the 24 weeks of follow-up (figure 1D, online supplemental figure S2), while no statistical significances were observed in ESR, complement C3 and C4, total IgG and its subclasses (online supplemental figure S3). However, there were four patients without response to telitacicept (improvement of IgG4-RI less than 2) during the 24 weeks of follow-up, resulting in a partial remission rate of 60% to telitacicept. Principal component analysis based on the baseline laboratory data distinguished the responsive and nonresponsive patients well on the first principal component (contributing rate: 56.97 %) (figure 1E). Analyses to the baseline data indicated that ESR, serum IgG4, IgG and plasmablast ratio contributed substantially to the status of ‘remission’, while total T, CD4^+^ T, CD8^+^ T and B cell counts contributed to the status of ‘non-remission’ (figure 1F and online supplemental table S3). This result suggested that patients with therapeutic response to telitacicept had relatively higher levels of serum immunoglobulin and plasmablast at baseline, while non-remission patients had relatively higher counts of lymphocytes.

To the best of our knowledge, this is the first study reporting the therapeutic potential of BLyS/APRIL-targeting biologics in IgG4-RD. Patients with IgG4-RD patients have limited treatment options, especially for those who were unable or unwilling to use long-term glucocorticoid therapy due to various conditions. Here, we reported a 60% partial remission rate at week 24, a duration widely adopted for the observation of IgG4-RD remission^7^ under the treatment of telitacicept, which showed potential effects on reducing lesion size, relieving symptoms and improving laboratory parameters in patients with IgG4-RD, especially in those who had high levels of ESR, IgG4, IgG and plasmablasts. Although future larger sample size studies are required to optimise the dosage and duration, this study may provide an important basis for developing a treatment strategy for IgG4-RD patients who are not suitable for glucocorticoid therapy.
