# Supplementary material for: BLyS/APRIL dual inhibition for IgG4-RD: a prospective single-arm clinical trial of telitacicept
Source: Ann Rheum Dis. 2023 Jan 19;82(6):881–3. doi: 10.1136/ard-2022-223529 (PMC10313947; doi:10.1136/ard-2022-223529)
Supplement: Supplementary data [file ard-2022-223529supp001.pdf]

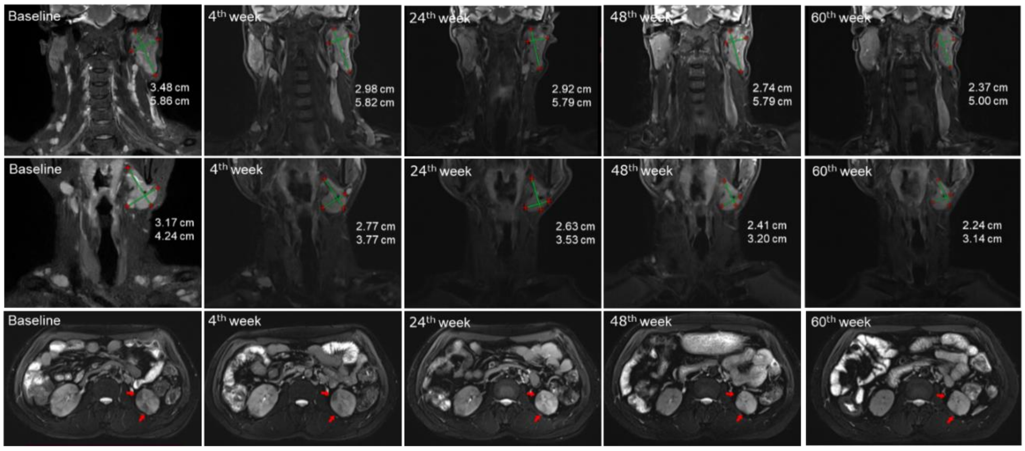

Figure S1 The maximum lesion area changes of multiple organs by MRI. The figure shows the maximum lesion area of left submandibular gland, bilateral parotid gland and lymph node in coronal plane, and the largest maximum lesion area of kidneys (red arrows) in transverse plane before (baseline) and after treatment with Telitacicept (at 4th, 24th, 48th and 60th week).

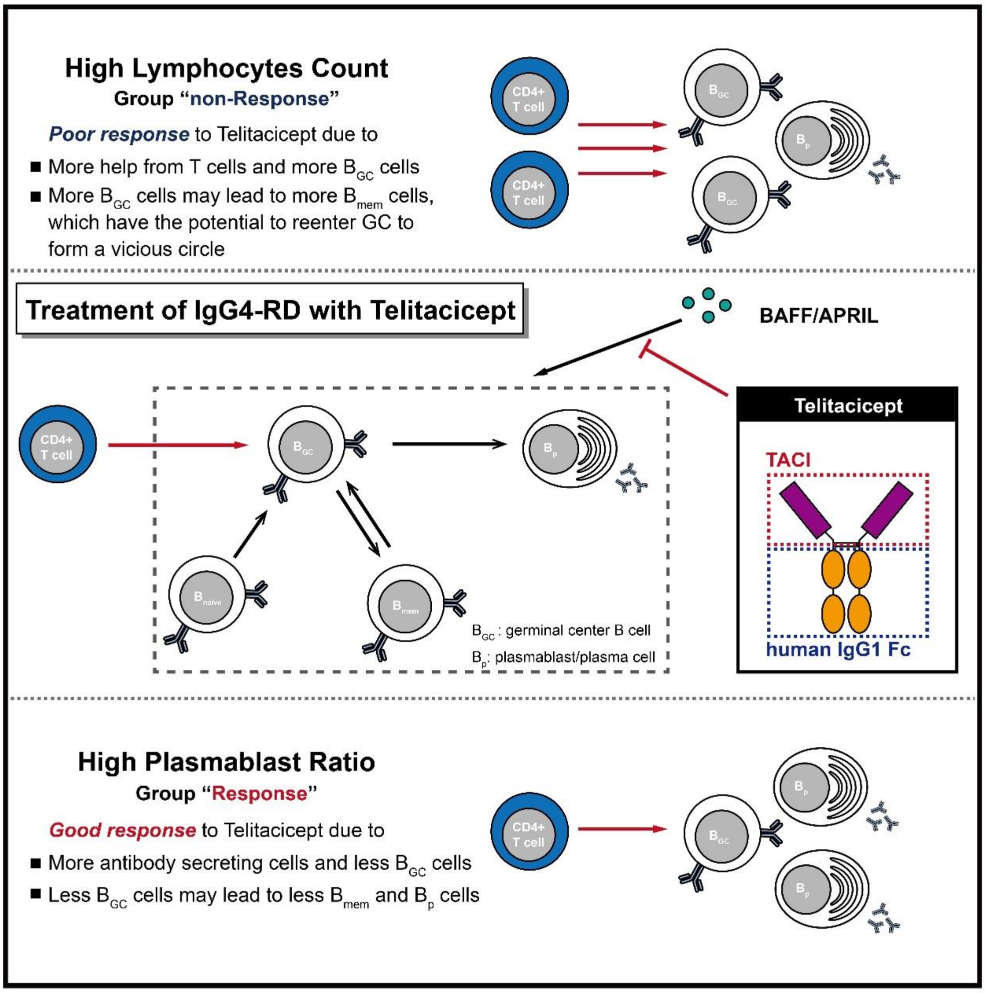

Figure S2 Treatment of IgG4-RD with Telitacicept, and an assumption of the dynamic of plasmablasts ratio observed in this clinical trial. For the plasmablasts' dynamic of IgG4-RD patients observed in Telitacicept treatment, although we didn't further investigate the detailed mechanism, some previous mechanistic studies related to BAFFR signaling may provide some clues: Zarnegar et al's study reveals that both canonical and non-canonical NF-κB signaling pathway can be activated by CD40L, while only non-canonical NF-κB signaling pathway can be stimulated by BAFF1; Lau et al's study shows that although BAFF/BAFFR signaling is important for the generation of germinal center independent, unmutated memory B cells, BAFFR is not essential for the survival and function of germinal center B cell and memory B cells that have undergone somatic hypermutations2; Guo, R. et al have also found that simultaneous activation of both BCR and CD40 was more potent in promoting B cell proliferation, when compared with the simultaneous activation with BCR and BAFFR3. Our recently published data also indicated a significant enrichment of germinal center reaction (GC reaction) related signatures in IgG4-RD involved tissues, and a large part of the B cells infiltrated into the involved tissue were germinal center B cell-like (revealed by single cell RNA-sequencing). GC reaction can promote the development

of memory B cells and plasma cells, and just like naïve B cells, memory B cells can reenter the germinal center and undergo further GC reactions4-6.

These clues indicated that BAFF may not be essential for the B cells in activated status, which relies more on the CD40-signaling. B cells in relatively "silent/steady" status have larger requirements for BAFF signaling. For the IgG4-RD patients, we assume: after receiving the treatment of Telitacicept, some activated B cells expanded and obtained (transformed to) the memory phenotype (influenced little by the BAFF/APRIL blockage, and therefore elevated in the early time after treatment), while the cells in relatively silent status (for the second/third/… wave attack) were "blocked" by Telitacicept, and thus cannot elicit a further autoimmune reaction, which leads to the disease remission and decrease of the corresponding cell subsets. Therefore, based on the aforementioned assumption, the ratio of plasmablasts elevated first, due to the potential unresponsiveness/poor responsiveness of already activated B cells to Telitacicept treatment, which further transformed/developed into plasmablasts, and decreased, due to the blockade of B cells that were not fully activated at the time of Telitacicept usage (if not intervened by Telitacicept, these not fully activated B cells could be further activated by pathogenic T cells).

References

1. Zarnegar B, He JQ, Oganesyan G, Hoffmann A, Baltimore D, Cheng G. Unique CD40-mediated biological program in B cell activation requires both type 1 and type 2 NF-kappaB activation pathways. Proc Natl Acad Sci U S A 2004;101:8108-13.
2. Lau AWY, Turner VM, Bourne K, Hermes JR, Chan TD, Brink R. BAFFR controls early memory B cell responses but is dispensable for germinal center function. J Exp Med 2021;218.
3. Guo R, Wang W, Yu L, Zhu Z, Tu P. Different regulatory effects of CD40 ligand and B-cell activating factor on the function of B cells. Int Immunopharmacol 2021;91:107337.
4. Gatto D, Brink R. The germinal center reaction. J Allergy Clin Immunol 2010;126:898-907; quiz 8-9.
5. Suan D, Sundling C, Brink R. Plasma cell and memory B cell differentiation from the germinal center. Curr Opin Immunol 2017;45:97-102.
6. Cai S, Chen Y, Hu Z, et al. The landscape of T and B lymphocytes interaction and synergistic effects of Th1 and Th2 type response in the involved tissue of IgG4-RD revealed by single cell transcriptome analysis. J Autoimmun 2022;133:102944.

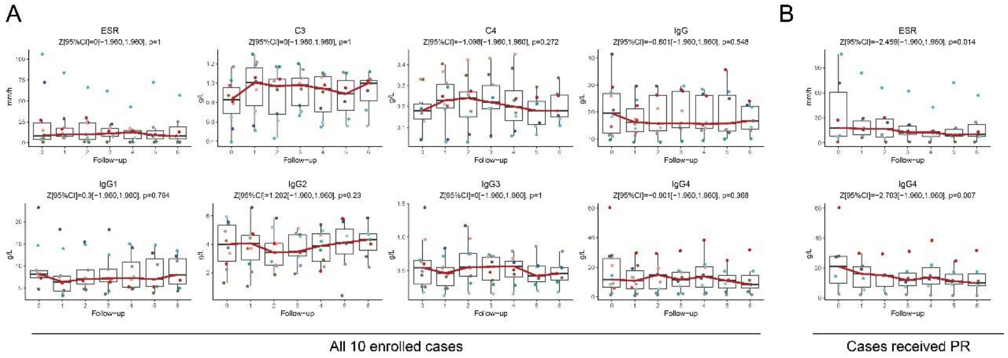

Figure S3 Trend analyses of other laboratory indices potentially reflecting the immune responses. A, Indices including ESR, C3, C4, IgG, and its subtypes (IgG1, IgG2, IgG3, and IgG4) were analyzed during the follow up period. **B**, Significant decreasing trends of ESR and serum IgG4 levels were observed in patients who received partial remission (PR) treated with Telitacicept.

Table S1 Inclusion and exclusion criteria

Inclusion Criteria
1. Patients to be included in the study must meet the classification criteria for IgG4-associated disease (2019ACR/EULAR);
2. Within half a year before enrollment, the patients did not use the following drugs:
A) Telitacicept or other biological agents with the same or similar targets;
B) Tumor necrosis factor inhibitor (TNFi) or tumor necrosis factor receptor antibody fusion protein;
C) Rituximab (or other B cell depleting agents);
3. Patients should be at least 18 years old at the time of enrollment;
4. Patients must agree to take a reliable contraceptive measure before enrolling;
5. The subject or his/her guardian agrees to participate in the study and signs the informed consent.
**Exclusion Criteria**
1. Currently pregnant or breastfeeding, or planning to become pregnant within half a year;
2. Significant health problems or diseases, including (but not limited to) the following: poorly controlled hypertension (>=160/95 mmHg), congestive heart failure (New York Heart Association Level III or IV), poorly controlled diabetes or functioning so poorly that they are unable to take care of themselves, other autoimmune diseases;
3. The total number of white blood cells <3,000/ µL, or platelets < 100,000/ µL, neutrophils < 1,500/ µL, or hemoglobin <8.5 g/ dL (85 g/L) during screening;
4. Active systemic infection (except common upper respiratory tract infection) occurred within 2 weeks before screening;
5. Current infection, chronic or recurrent infectious disease, or evidence of latent tuberculosis infection (as determined by PPD or T-SPOT);
6. Known to be infected with HIV, hepatitis B, or hepatitis C at screening time;
7. A history of lymphoproliferative disease or any known malignancy or a history of malignancy in any organ system within the last 5 years;
8. Allergic to Telitacicept;
9. Participating in other clinical trials;
10. Any medical or psychiatric condition that the Investigator considers will prevent participants from following the protocol or completing the study according to the protocol;
11. Patients refuse to comply with the requirements of this study to complete the study;
12. Any other circumstances considered by the Investigator to be inappropriate for participation in this study.

Table S2 Characteristics of enrolled cases

ID	Gender	Involved Organ	Baseline IgG4-RD RI (2012)	Endpoint IgG4-RD RI (2012)	Response to Telitacicept	History	Discontinuation of the glucocorticoids in the 1st week of treatment
1	Female	Lacrimal glands,submandibular glands,kidneys,lymph nodes	15	14[#]	No	Treatment naïve	0.6 mg/kg methylprednisolone for 7 days
2	Male	Submandibular glands,lymph nodes	9	6	Yes	Treatment naïve	0.6 mg/kg methylprednisolone for 7 days
3	Male	Submandibular glands,parotid glands,lymph nodes	9	8	No	Relapse, no medication usage for 6 months	0.6 mg/kg methylprednisolone for 7 days
4	Male	Submandibular glands,parotid glands,kidneys,lymph nodes	12	4	Yes	Relapse, no medication usage for 18 months	0.6 mg/kg methylprednisolone for 7 days
5	Male	Lacrimal glands,musculus ocularis,submandibular glands,lung, retroperitoneum,lymph nodes	18	24	No	Relapse, no medication usage for 3 months	0.6 mg/kg methylprednisolone for 7 days
6	Male	Lacrimal glands,submandibular glands, retroperitoneum,lymph nodes	15	9	Yes	Treatment naïve	0.6 mg/kg methylprednisolone for 7 days
7	Male	Pancreas,lacrimal glands,submandibular glands,musculus ocularis,lymph nodes	15	5	Yes	Treatment naïve	0.6 mg/kg methylprednisolone for 7 days
8	Male	Lacrimal glands,submandibular glands,parotid glands,lymph nodes	12	7	Yes	Treatment naïve	0.6 mg/kg methylprednisolone for 7 days
9	Male	Lacrimal glands,submandibular glands,parotid glands,lymph nodes	12	4	Yes	Relapse, no medication usage for 18 months	0.6 mg/kg methylprednisolone for 7 days
10	Female	Lacrimal glands,submandibular glands,parotid glands,lymph nodes	12	16	No	Treatment naïve	0.6 mg/kg methylprednisolone for 7 days

Note: there is no statistical difference between group "non-Response" and "Response" in index "**Number of involved organ**" ("non-Response" vs. "Response", 4.00[3.25,5.50] vs 4.00[3.50,4.25], p=0.904) and "**Baseline IgG4-RD RI (2012)**" ("non-Response" vs. "Response", 13.50[9.75,17.25] vs 12.00[11.25,15.00], p=0.737). All the aforementioned data are presented as median[IQR], and analyzed with Mann-Whitney U test. The "**Baseline IgG4-RD RI (2012)**" evaluations were based on reference [Carruthers MN, Stone JH, Deshpande V, Khosroshahi A. Development of an IgG4-RD Responder Index. International journal of rheumatology 2012;2012:259408.]. # addition of glucocorticoids in the 5th week without authorization based on the progressive lacrimal gland enlargement and elevated serum IgG4 level (disease relapse). Therefore, this patient discontinue the trial, and the IgG4-RD RI recorded her status at that time-point. The median age of patients at the time of screen was 51.5 years old, ranged from 25 to 71.

Table S3 Baseline parameters in remission and non-remission patients after Telitacicept treatment.

	Group		Estimated differences, Non-remission vs Remission [95% CI]	*p* value
	Non-remission (n=4)	Remission (n=6)		
T cell (/μL)	2023[1748,2471]	733[682,800]	1279[357,2912]	0.0095
CD4+T cell (/μL)	1121[1011,1623]	528[424,574]	634[338,2411]	0.0095
B cell (/μL)	268[236,300]	114[76,125]	152[75,230]	0.0095
L# ($\times10^9$/L)	2.93[1.882,4.358]	1.32[1.158,1.46]	1.645[0.19,4.55]	0.019
CD45RA-CCR7- ($\times10^4$ /mL)	74.945[64.49,113.401]	24.577[14.064,29.396]	50.408[16.309,194.433]	0.019
CD27+IgD- ($\times10^4$ /mL)	3.797[2.352,5.355]	1.097[0.836,1.511]	2.583[0.005,5.847]	0.0381
CD27+IgD+ ($\times10^4$ /mL)	3.496[2.596,4.105]	0.605[0.379,0.796]	2.905[0.14,4.478]	0.0381
CD45RA+CCR7- ($\times10^4$ /mL)	8.916[7.595,10.312]	4.878[3.745,6.239]	4.038[0.57,7.504]	0.0381
CD27+ plasmablasts (%)	0.49[0.292,0.76]	1.21[0.868,1.605]	-0.68[-2.88,0.05]	0.0667
CD27-IgD+ ($\times10^4$ /mL)	17.625[15.285,18.482]	7.419[4.408,8.808]	9.668[-0.263,14.341]	0.0667
C4 (g/L)	0.205[0.188,0.248]	0.15[0.062,0.178]	0.066[-0.11,0.2]	0.1066
IgG (g/L)	12.8[10.707,17.407]	23.97[18.882,28.727]	-9.13[-26.89,5.2]	0.1143
IFN-γ (pg/mL)	0.93[0.7,1.37]	2.07[1.517,2.825]	-1.32[-5.68,0.97]	0.1143
CD8+T cell (/μL)	609[486,697]	254[192,298]	355[-69,636]	0.1143
CD20+BCMA+TACI+ (%)	3.23[2.91,3.517]	1.435[1.322,2.53]	1.51[-0.15,2.78]	0.1143
CD20+BCMA+TACI- (%)	3.005[2.713,3.84]	2.43[2.365,2.638]	0.57[-1.23,3.26]	0.1143
CD24-plasmablasts (%)	1.63[1.138,1.985]	3.035[1.8,4.818]	-1.68[-4.46,0.37]	0.1143
CD45RA-CD25+ ($\times10^4$ /mL)	19.504[13.508,49.198]	10.56[4.87,16.031]	10.18[-6.158,111.978]	0.1143
Baso# ($\times10^9$/L)	0.06[0.043,0.073]	0.025[0.013,0.038]	0.03[-0.01,0.06]	0.1332
Mono# ($\times10^9$/L)	0.59[0.495,0.742]	0.375[0.363,0.418]	0.204[-0.02,0.68]	0.1344
IL-4 (pg/mL)	1.515[1.402,1.888]	2.545[1.83,3.035]	-0.586[-1.74,0.35]	0.1344
CD27-IgD- ($\times10^4$ /mL)	3.872[1.977,5.539]	1.071[0.701,1.198]	2.096[-0.455,5.134]	0.1714
ESR (mm/h)	4.5[3.25,7.5]	18[8.25,60.75]	-8.09[-101,6]	0.1995
